# *Escherichia coli* and *Pseudomonas aeruginosa* Isolated From Urine of Healthy Bovine Have Potential as Emerging Human and Bovine Pathogens

**DOI:** 10.3389/fmicb.2022.764760

**Published:** 2022-03-07

**Authors:** Silvia Giannattasio-Ferraz, Adriana Ene, Vitor Júnio Gomes, Cid Oliveira Queiroz, Laura Maskeri, André Penido Oliveira, Catherine Putonti, Edel F. Barbosa-Stancioli

**Affiliations:** ^1^Departamento de Microbiologia, Instituto de Ciências Biológicas, Universidade Federal de Minas Gerais, Belo Horizonte, Brazil; ^2^Bioinformatics Program, Loyola University Chicago, Chicago, IL, United States; ^3^Empresa de Pesquisa Agropecuária de Minas Gerais – EPAMIG, Uberaba, Brazil; ^4^Department of Biology, Loyola University Chicago, Chicago, IL, United States; ^5^Department of Microbiology and Immunology, Stritch School of Medicine, Loyola University Chicago, Maywood, IL, United States

**Keywords:** microbiota, prophage, *Escherichia coli*, *Pseudomonas aeruginosa*, bovine, urine, emerging pathogens

## Abstract

The study of livestock microbiota has immediate benefits for animal health as well as mitigating food contamination and emerging pathogens. While prior research has indicated the gastrointestinal tract of cattle as the source for many zoonoses, including Shiga-toxin producing *Escherichia coli* and antibiotic resistant bacteria, the bovine urinary tract microbiota has yet to be thoroughly investigated. Here, we describe 5 *E. coli* and 4 *Pseudomonas aeruginosa* strains isolated from urine of dairy Gyr cattle. While both species are typically associated with urinary tract infections and mastitis, all of the animals sampled were healthy. The bovine urinary strains were compared to *E. coli* and *P. aeruginosa* isolates from other bovine samples as well as human urinary samples. While the bovine urinary *E. coli* isolates had genomic similarity to isolates from the gastrointestinal tract of cattle and other agricultural animals, the bovine urinary *P. aeruginosa* strains were most similar to human isolates suggesting niche adaptation rather than host adaptation. Examination of prophages harbored by these bovine isolates revealed similarity with prophages within distantly related *E. coli* and *P. aeruginosa* isolates from the human urinary tract. This suggests that related urinary phages may persist and/or be shared between mammals. Future studies of the bovine urinary microbiota are needed to ascertain if *E. coli* and *P. aeruginosa* are resident members of this niche and/or possible sources for emerging pathogens in humans.

## Introduction

While the urinary tract (UT) of healthy individuals was traditionally believed to be sterile, we now know that bacteria do persist within the UT of healthy humans, albeit at a lower biomass than other areas of the human body (Dong et al., 2011; Wolfe et al., 2012). Furthermore, bacteriophages (viruses that infect bacteria) are abundant within the healthy human UT (Santiago-Rodriguez et al., 2015; Miller-Ensminger et al., 2018), and likely play a role in modulating the diversity and relative abundance of bacteria within the community (Garretto et al., 2019). Understanding the urinary microbiota of healthy individuals has been instrumental in contributing to our understanding of UT symptoms and disease in humans [see reviews (Whiteside et al., 2015; Neugent et al., 2020)]. More recently, microbiome studies have found bacteria within the UT of other healthy mammals, including canines (Burton et al., 2017) and swine (Torres Luque et al., 2020). Furthermore, through our prior efforts, we have isolated several different bacterial taxa from the UT of healthy cattle (Giannattasio-Ferraz et al., 2020a,b,c,d,e, 2021a).

A common cause of urinary tract infections (UTIs) in mammals is *Escherichia coli* (Wooley and Blue, 1976; Yeruham et al., 2006; Foxman, 2014; Moreno et al., 2018). In cattle, *E. coli* as well as *Corynebacterium renale*, *Streptococcus* spp., *Proteus* spp. *Klebsiella* spp., and *Pseudomonas aeruginosa* are common causes of UTIs, and it is believed that these uropathogens are frequently introduced from the vaginal tract (Yeruham et al., 2006). Prior microbiome surveys have found both *E. coli* and *P. aeruginosa* within the vagina of healthy cows (Padula and Macmillan, 2006; Laguardia-Nascimento et al., 2015; Giannattasio-Ferraz et al., 2019). In cattle, bacterial infections related to the urogenital tract are responsible for exorbitant losses in sectors such as reproduction and the dairy industry (Yeruham et al., 2006; Sheldon et al., 2008). Furthermore, inflammation of the reproductive tract, causing endometriosis in previously healthy cows, is a common cause of infertility (Williams et al., 2008). Increasing levels of antibiotic resistance within dairy animals is of paramount concern for livestock quality of life, potential spread to humans, and economic impact (Sharma et al., 2017).

Given the significant impact of UTIs on the dairy industry, we initiated an investigation of two uropathogenic species, *E. coli* and *P. aeruginosa*, in healthy animals. In addition to their impact on urinary health, these two species also are frequent causes of mastitis (Banerjee et al., 2017; Lavon et al., 2019). However, as we found, both taxa can also be found within the UT of healthy cows. This parallels recent observations in the human female UT – uropathogens reside within the UT microbiota of healthy individuals (Thomas-White K. J. et al., 2018; Garretto et al., 2020; Price et al., 2020). The presence of uropathogens in the healthy urinary tract suggests that dysbiosis rather than introduction of uropathogens from external sources is the cause of infection (Thänert et al., 2019; Garretto et al., 2020). Here, we describe 5 *E. coli* and 4 *P. aeruginosa* strains isolated from urine of healthy Gyr cattle, an important dairy breed in Brazil. To date, these are the first genomes for urinary isolates of these two species from healthy bovines. We compared these strains to bovine isolates from other niches and human urinary genomes as well as their resident prophages in an effort to explore niche vs. host adaptations.

## Materials and Methods

### Animals and Sample Collection

For this study, we isolated 5 *E. coli* strains and 4 *P. aeruginosa* from urine collected from healthy Gyr heifers at the Agricultural Research Company of Minas Gerais State (EPAMIG) in Brazil. In total urine samples were collected from 10 different heifers in this herd. The reproduction within the herd is controlled by fixed time artificial insemination and there is no presence of bulls. This herd has a medium milk production of 3,700 lg/lactation/cow and a rigorous sanitary control. None of the animals from this study presented clinical reproductive signs for 12 months before sample collection. The sample collection and following experiments were previously approved by the Ethics Committee in Animal Experimentation of the Universidade Federal de Minas Gerais, Brazil (CEUA/UFMG - 40/2019). Prior to the urine sampling, the external genitalia of the animals were cleaned with soap and water. The mid-stream urine was collected using a sterile 50 ml centrifuge tube, frozen (−20°C), transported to the lab and processed. All samples were processed within 48 h of sampling.

### Bacterial Isolation and Identification

Four of the urine samples were processed using the same technique as follows. First, samples were aliquoted and spun down. 500 μL of the supernatant was spread on an LB agar plate, incubated overnight at 37°C and the individual colonies were picked. The colonies were regrown in LB agar overnight at 37°C and this process was repeated at least 3 times to obtain pure colonies. The pure single colonies were grown in liquid LB media overnight at 37°C. Samples were spun down and the DNA was extracted using the Qiagen DNeasy UltraClean Microbial Kit following the manufacturer’s protocol. All of the isolates were submitted to sequencing of the 16S rRNA region using the 63F/1387R primer pair to identification. Sanger sequencing was performed by Genewiz (New Brunswick, NJ, United States) using each primer individually, providing 2x coverage. Raw reads were then manually trimmed, assembled and queried against the NCBI 16S rRNA Sequences Database via the blastn algorithm. Based upon the 16S rRNA gene sequence, four isolates from four different animals were identified as *P. aeruginosa* and five isolates, from the same four animals, were identified as *E. coli*. Isolates of other taxa have been described elsewhere (Giannattasio-Ferraz et al., 2020a,b,c,d,e, 2021a,b).

### Whole Genome Sequencing, Assembly and Characterization

Whole genome sequencing was performed for all 9 isolates. The extracted DNA was sequenced at the Microbial Genomic Sequencing Center (MiGS) (Pittsburgh, PA, United States). For sequencing, the libraries were prepared using the Illumina Nextera kit and the genomes were sequenced using the NextSeq 550 platform. Raw reads were trimmed using Sickle v1.33^1^ with a quality threshold (Phred quality score = 20) and length threshold after trimming of 100 nucleotides. Trimmed reads were then assembled using SPAdes v3.13.0 with the “only assembler” option for k values of 55, 77, 99, and 127 (Bankevich et al., 2012). To calculate genome coverage for the assemblies, BBMap v38.47^2^ was used. The raw reads and genome assemblies were deposited in GenBank, Table 1 shows the accession number for each BioSample deposited. The assembled genomes were annotated using the NCBI Prokaryotic Genome Annotation Pipeline (PGAP) v4.11 (Tatusova et al., 2016).

**TABLE 1:** Genome statistics for the isolated *Escherichia coli* and *Pseudomonas aeruginosa.*

Species	Strain	BioSample Accession #	Genome Coverage	GC Content (%)	# Contigs	N50	# Coding Genes	# tRNAs	CheckM (contamination, completeness)
*E. coli*	UFMG-H6A	SAMN14470510	61	44.36	49		4,213	76	0%, 100%
	UFMG-H7A	SAMN14470512	40	45.32	50		4,209	73	0%, 100%
	UFMG-H7C	SAMN14486423	57	50.48	59		4,251	71	0.5%, 93.3%
	UFMG-H9	SAMN14470514	44	45.69	50		4,22	75	0%, 100%
	UFMG-H10	SAMN14470515	82	49.19	43		4,217	75	0%, 100%
*P. aeruginosa*	UFMG-H6	SAMN14470516	104	64.68	49		5,698	57	0.6%, 99.3%
	UFMG-H7	SAMN14470517	103	64.43	47		5,762	57	0.6%, 99.3%
	UFMG-H9	SAMN14470518	95	62.98	51		5,644	57	0.6%, 98.6%
	UFMG-H10	SAMN14470519	112	64.21	47		5,674	57	0.6%, 99.1%

Genome assemblies also were examined with the Center for Genomic Epidemiology’s (CGE) tools PlasmidFinder v2.1 (Carattoli et al., 2014), using the Enterobacteriaceae database and a 90% threshold for identity and 60% minimum coverage, and ResFinder v3.2 (Zankari et al., 2012) with default parameters. The serotype for *E. coli* strains was verified using CGE’s SerotypeFinder v2.0 (Joensen et al., 2015). *P. aeruginosa* serotypes were determined using CGE’s Past v1.0 (Thrane et al., 2016). *E. coli* phylotype were determined using EzClermont (Waters et al., 2020). The VFAnalyzer tool was used to predict virulence factors (Liu et al., 2019). All of the strains were also screened for the CRISPR/Cas system (Grissa et al., 2007). Genes for flagellar synthesis, flagellar rotation, chemotactic signal transduction, and chemotactic membrane receptors were identified within the genome sequences using reciprocal blasts and manual curation. Local blast databases were created with the PGAP annotation files of the bovine urinary strains; blastn queries were used to identify the presence of *flg* (*flgA*-*flgN*), *flh* (*flhA*-*flhE*), *fli* (*fliA*, *fliC*-*fliT*, and *fliZ*), *mot* (*motA*, *motB*) and *che* (*cheA*, *cheB*, *cheR*, *cheW*, *cheY*, and *cheZ*) genes as well as chemotactic receptors *tap*, *tar*, *trg*, *tsr*, and *aer*. These sequences were manually inspected. Unless previously noted, default parameters were used for each tool.

### Comparative Genomics

The 5 bovine urinary *E. coli* and 4 bovine urinary *P. aeruginosa* strains were first compared to bovine isolates from other niches (Supplementary Table 1). In the case of *E. coli*, 3049 bovine genomes are publicly available; the vast majority are from fecal samples (*n* = 1199). A subsample of these genomes were selected, limiting genomes to those isolated (regardless of sample type) from South America or vaginal samples [given prior evidence that uropathogens are frequently introduced from the vaginal tract (Yeruham et al., 2006)]. All bovine *P. aeruginosa* genomes were considered (*n* = 20). Genomes were retrieved from NCBI and compared to the bovine urinary genomes by ANI (Average Nucleotide Identity) using pyANI and the ANIm measure (Pritchard et al., 2016). Shared genic content was determined via reciprocal blastn queries. 95% nucleotide sequence identity was used as the threshold for these blasts, which were conducted locally using the blast + executable (v2.9.0). These other bovine isolates were also characterized using CGE’s SerotypeFinder v2.0 (Joensen et al., 2015), CGE’s Past v1.0 (Thrane et al., 2016), and EzClermont (Waters et al., 2020), as described above.

A comparative analysis was performed using publicly available genomes and genome assemblies of isolates from human urine. Urinary isolates were identified by referencing GenBank sequencing metadata (isolation source). Only quality assemblies were considered, based upon their completeness and checkM contamination score through PATRIC (Davis et al., 2020). The 5 bovine *E. coli* isolates were compared to 900 human urinary *E. coli* genomes/assemblies and the 4 bovine *P. aeruginosa* isolates were compared to 221 human urinary *P. aeruginosa* genomes/assemblies. The strains retrieved and their accession numbers are listed in Supplementary Table 2. The genomes were used to identify the pangenome of both species using anvi’o v6.2 (Eren et al., 2015). The pangenome was created using the anvi-pan-genome with mcl-inflation 10 and the other parameters were default. The concatenated core gene sequences were found using the anvi-get-sequences-for-gene-clusters command. Whole genome comparisons were conducted using the Mash program (Ondov et al., 2016) with default *k* and sample size (*s*) of 1,000,000.

The phylogenetic trees were derived using FastTree v2.1.11 (Price et al., 2010) plug-in through Geneious Prime using default parameters. Resulting trees were then visualized using iTOL (Letunic and Bork, 2016).

### Prophage Prediction

All *E. coli* and *P. aeruginosa* urinary genomes/assemblies included in our comparative genomics analysis (Supplementary Table 2) were uploaded to the tool PHASTER for prophage prediction (Arndt et al., 2016). PHASTER predicts incomplete, questionable and intact prophage regions. Only intact regions were used for our analysis. The intact nucleotide sequences were queried against NCBI nr/nt database’s viral sequences (TaxID: 10239) to predict the taxonomies of the prophages. GraphPad Prism v8.8.1 was used to illustrate taxonomic predictions of prophages found. Homologous gene sequences between predicted phages were identified anvi’o v6.2 (Eren et al., 2015). Gene calls were made using anvi’o and homologous proteins were identified with a minbit of 0.35. This choice of threshold is informed by prior work identifying homologous phage proteins [see (Shapiro and Putonti, 2021)]. These homologous gene clusters were then processed using a python script written to generate a gene presence/absence matrix and an edge list. This script is available through our GitHub repository.^3^ The resulting edge list was visualized using Cytoscape v3.8.2 (Shannon et al., 2003).

## Results

### Bovine Urinary Tract *Escherichia coli* and *Pseudomonas aeruginosa* Genome Characterization

We isolated 5 *E. coli* strains and 4 *P. aeruginosa* from the urine of 4 healthy Gyr heifers and sequenced their genomes (Table 1). For each animal, both *E. coli* and *P. aeruginosa* were isolated; 2 *E. coli* strains were isolated from the same individual (UFMG-H7A and UFMG-H7C). All 5 *E. coli* genomes are characterized by the H34 flagellar antigen and are representatives of the phylotype B1, and all 4 *P. aeruginosa* genomes belong to the O5 serotype. Sequencing did not reveal the presence of plasmids within any of the strains. The CRISPR/Cas system was identified in all 5 of the *E. coli* genomes (type I-E). Furthermore, all 5 *E. coli* genome assemblies were found to include fimbriae (Supplementary Table 3) as well as genes for flagellar synthesis, flagellar rotation, chemotactic signal transduction, and chemotactic membrane receptors. Antibiotic resistance genes were only detected in the *P. aeruginosa* genome assemblies; all 4 *P. aeruginosa* strains are predicted to have the same resistances Phenicol (*catB7*), Beta-lactam (*bla*_OXA–50_ and *bla*_PAO_), Fosfomycin (*fosA*), and Aminoglycoside [*aph*(3′)-IIb].

### Comparative Genomics

The bovine urinary *E. coli* and *P. aeruginosa* genomes were first compared to bovine isolates via ANI. These genomes include isolates from fecal, milk, nasopharynx, vaginal, and internal organs. As expected, high ANI values were observed for these different strains of the same species (Supplementary Figure 1). Of note, the bovine urinary *E. coli* and *P. aeruginosa* genomes clustered distinctly from genomes from other isolation sites. Shared genic content between the bovine urinary genomes ranged from 63.46 to 93.25% for the bovine *E. coli* isolates and from 78.51 to 96.17% for the bovine *P. aeruginosa* isolates (Supplementary Table 1). The bovine urinary *E. coli* strains are representatives of the B1 phylotype; strains from bovine fecal and vaginal samples are also associated with this phylotype (Supplementary Table 1). In addition to the bovine urinary *P. aeruginosa* strains, genomes from bovine nasopharynx and milk isolates are also representatives of the O5 serotype.

Next, we compared the bovine urinary *E. coli* and *P. aeruginosa* genomes to all publicly available genomes of human urinary isolates. The pangenome and single copy number core genome was identified for 905 urinary *E. coli* strains (inclusive of the 5 bovine *E. coli* strains sequenced here) and for the 225 urinary *P. aeruginosa* strains (inclusive of the 4 bovine *P. aeruginosa* strains sequenced here).

**FIGURE 1:**
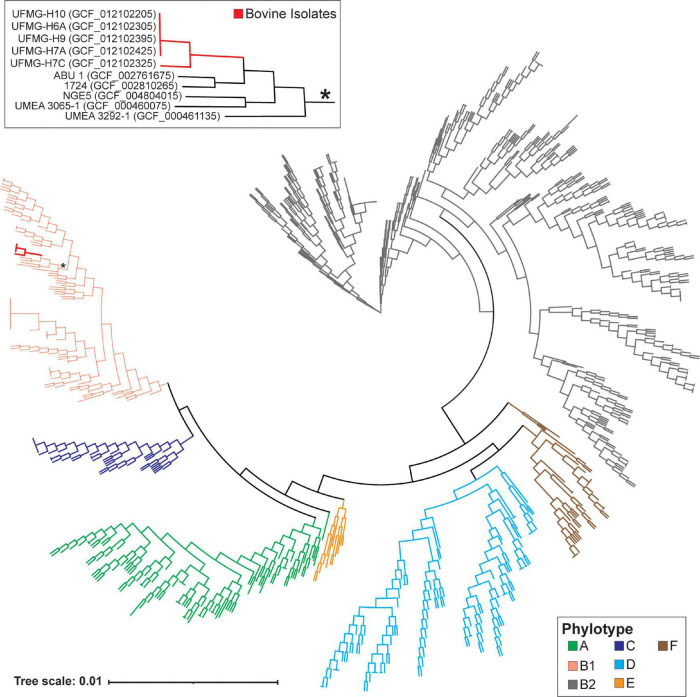
Core phylogenomic tree of bovine and human urinary isolates of *Escherichia coli*. This tree was generated from the sequence alignment of the amino acid sequences for the 346 identified single copy core genes (see Methods). Bovine isolates are indicated in red, and the clade containing these isolates is indicated by an asterisk and shown in the insert. The phylotype for the genomes is indicated by the branch color.

The analysis of the 900 human and 5 bovine urinary *E. coli* isolates identified 23,678 homologous genes. The majority (75.47%) of the *E. coli* genomes contained one or more genes that were unique to their genome, i.e., they were not present in any of the other urinary *E. coli* genomes examined here. UFMG-H7C included 201 genes that were unique to its genome, while the other 4 bovine isolates had no unique genes. Given the diversity in gene content observed among the 905 urinary *E. coli* genomes in this pangenome, a small single copy gene core genome was identified, which had 346 genes. Using this core, a phylogenomic tree for the *E. coli* strains was derived (Figure 1). This tree places the 5 bovine urinary isolates (shown in bold red) in a single clade (red), within the B1 phylotype. These bovine urinary strains have a core genome most similar to human isolates from individuals with either asymptomatic bacteriuria (strains: ABU_1) or UTI (strains: 1724, NGE5, UMEA 3065-1, UMEA 3292-1), per GenBank metadata.

**FIGURE 2:**
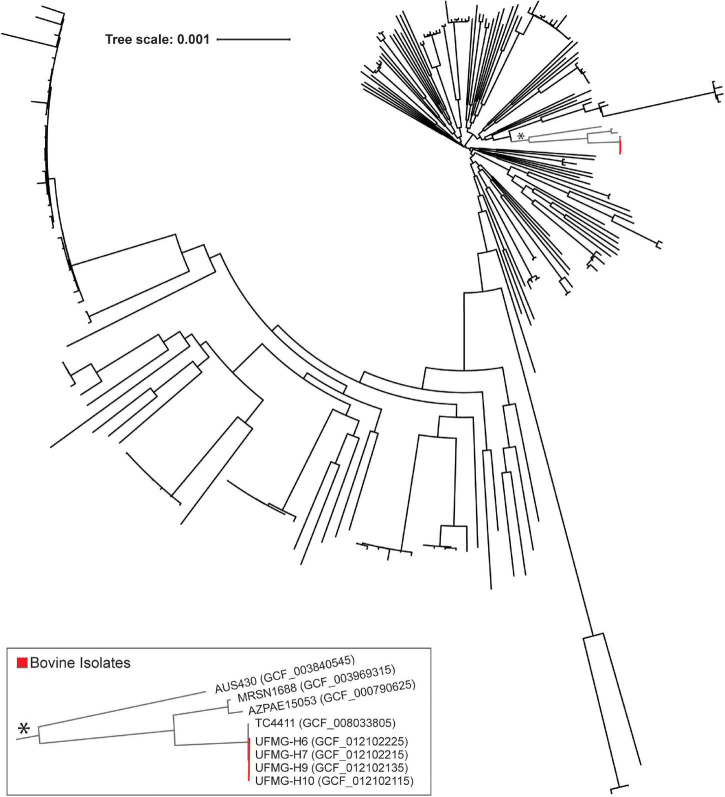
Core phylogenomic tree of bovine and human urinary isolates of *Pseudomonas aeruginosa*. This tree was generated from the sequence alignment of the amino acid sequences for the 1,546 identified single copy core genes (see Methods). Bovine isolates are indicated in red, and the clade containing these isolates is indicated by an asterisk and shown in the insert.

The pangenome and core genome also was computed for the 225 urinary *P. aeruginosa* genomes. 19,257 homologous genes were identified in the genomes, with 96.89% of these genomes containing at least one gene sequence unique amongst the group. Nevertheless, 1,546 of the homologous genes are single copy core gene sequences, shared amongst all urinary *P. aeruginosa* genomes. The phylogenomic tree of these core genes places the 4 bovine *P. aeruginosa* isolates together (Figure 2, red). The bovine clade’s core genome is most similar to that of strain TC4411 (Accession No. GCF_008033805), collected from human urine in Besancon, France in 2017; however, the donor’s symptom status is unknown. Other closely related genomes include those isolated from individuals with UTI (strains: AZPAE15053 and AUS430) or symptom status unknown (strain: MRSN1688).

Last, we investigated if the bovine urinary genomes were more similar to other bovine isolates or to human urinary isolates. The 5 bovine urinary *E. coli* genomes were compared to all of the bovine genomes previously considered in our ANI analysis (Supplementary Table 1) and the human urinary isolates most closely related to the bovine urinary genomes (genomes in the insert of Figure 1). The most closely related genome sequence for the 5 bovine urinary isolates was the human urinary isolate *E. coli* ABU 1. On average, the bovine urinary isolates were as similar to the human urinary isolates (n = 900) as the bovine vaginal isolates (*n* = 9) (Supplementary Table 4). The bovine urinary *P. aeruginosa* genomes were most similar to the human urinary genome strain TC4411 and more similar to the human urinary genomes than the genomes isolated from other bovine samples (Supplementary Table 4).

### Phage Prediction

**FIGURE 3:**
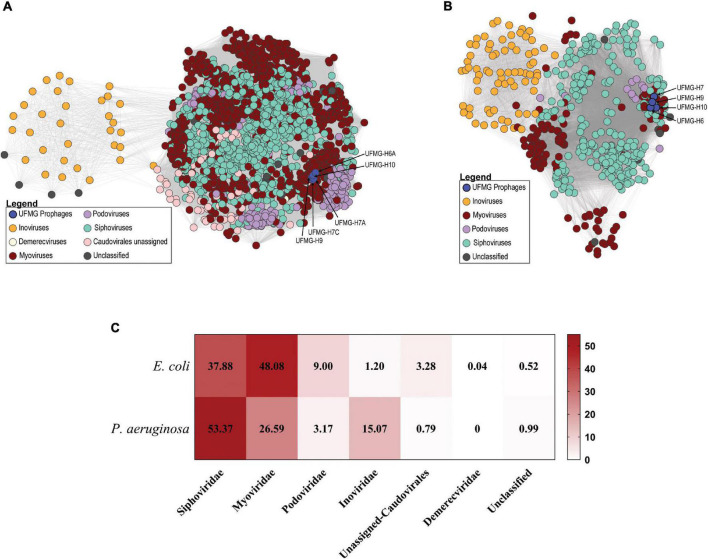
Similarity of bovine and human urinary prophages. **(A)** Predicted prophages from *Escherichia coli* analyzed strains. Nodes represent prophages and two nodes are connected if they encode for a homologous gene (gray line; edge). The prophages from the bovine urinary strains are highlighted in blue and are predicted to belong to the *Myoviridae* family. **(B)** Predicted prophages from *Pseudomonas aeruginosa* analyzed strains. Again, nodes represent prophages, and two prophages are connected if they encode for a homologous gene (gray line; edge). The prophages from the bovine urinary strains are highlighted in blue (unassigned *Caudovirales*). **(C)** Comparison of percentage of predicted prophages of *E. coli* and *P. aeruginosa*.

Prophage prediction was also conducted for all of the human urinary genomes and the bovine urinary genomes (Supplementary Table 3). Only high confidence (predicted to be intact) prophages were considered for downstream analysis. A total of 2,663 prophages were identified. Sequence similarity to characterized phage genomes enables us to assign all but 14 of these prophage sequences to one of 6 different viral families (Figure 3C); 14 predicted prophages exhibited no sequence similarity to any characterized phages (“Unclassified”).

The 5 *E. coli* UFMG strains include one intact prophage sequence each, which are identical at the nucleotide level and are 40.1 kb long, encoding for 51 viral proteins including tail and head associated proteins, repressor proteins and integrase. The bovine prophages exhibit greatest sequence homology (61% query coverage and 97% sequence identity) to the myovirus *Shigella* phage SfII (Accession No. NC_021857). The bovine prophages exhibited sequence similarity to prophages identified within the human urinary *E. coli* genomes. They shared genes, and thus are connected in Figure 3A, to 1,767 human urinary prophages. The number of shared genes ranged from 1 to 46. We further investigated human urinary prophages that shared over 80% of their genes with the bovine urinary prophages. Seven prophages met this threshold, harbored by the following *E. coli* strains: 21_fCAUTI, HM46, UMB1346, UMB1347, UMB1354, UMB1359, and UMB5337. Sequence alignment found that these sequences had 72-84% nucleotide sequence identity to the bovine urinary prophages; the five UMB strains are identical. Referring to the core phylogeny of the urinary core genome identified the bacterial strains containing these prophages were phylogenetically distant to the 5 bovine urinary *E. coli* strains (Supplementary Figure 2).

Among the *P. aeruginosa* strains analyzed, 495 phages were predicted belonging to 4 different viral families; nine predicted prophages exhibited no sequence homology to characterized phages (“Unclassified”) (Figure 3B). Each of the bovine *P. aeruginosa* strains contained one predicted intact prophage, which were identical (100% nucleotide sequence identity). This prophage is 34.9 kb long (63.96% GC content) with 42 viral proteins, including an excisionase, two hydrolases and tail associated proteins. While identical across the bovine urinary strains, these prophage sequences had very little sequence similarity to a previously characterized phage [3% query coverage and 76% sequence identity to *Pseudomonas* phage vB_Pae_BR141a (Accession No. MK510991.1)]. Given the placement of the bovine *P. aeruginosa* prophages in Figure 3B, the bovine phages are predicted to belong to the order *Caudovirales*.

Homologs to the bovine urinary prophage also were identified in the human urinary *P. aeruginosa* genomes. The bovine urinary prophage encoded genes homologous with 224 prophages in human urinary strains, ranging from a single similar gene to all 42 annotated genes. In fact, the bovine urinary prophage sequence was identical to a prophage harbored by *P. aeruginosa* TC4411 (Accession No. GCF_008033800), the same strain that the bovine urinary core genomes claded with in Figure 2. It also exhibited over 98% sequence identity with 10 other human urinary *P. aeruginosa* strains. These prophages were found in *P. aeruginosa* strains closely related to the bovine urinary isolates, as well as more distantly related *P. aeruginosa* strains (Supplementary Figure 3).

Most of the prophages identified for the two species are predicted to be tailed phages (order: *Caudovirales*) (Figure 3C). The predicted prophages are most frequently predicted to be representatives of *Siphoviridae* and *Myoviridae*, 85.96% and 79.96%, respectively. For the two bacterial hosts, however, the *Siphoviridae* presented higher percentage numbers in the urinary *P. aeruginosa* strains in comparison to *Myoviridae* in *E. coli*. Furthermore, *Inoviridae* (order: *Tubuvirales*) were detected more frequently in the urinary *P. aeruginosa* strains and *Podoviridae* in the *E. coli* strains.

## Discussion

The human UT microbiota is the best studied UT microbiota amongst mammals. Isolation of strains from the bovine UT, including our prior work investigating other bacterial taxa (Giannattasio-Ferraz et al., 2020a,b,c,d,e, 2021a) and our study presented here, is a further step toward understanding the healthy bovine UT microbiota. Furthermore, here we have compared these bovine isolates to human UT isolates, producing the first examination of urinary bacteria and urinary phages from two different mammals.

*Escherichia coli* and *P. aeruginosa* are frequently pathogenic species for cattle. *E. coli* variants in the vagina have been associated with post-partum uterine diseases in cattle, resulting in significant economic losses in agriculture (Moreno et al., 2020), and within the UT; *E. coli* can cause UTIs (McKinnon et al., 2018). Furthermore, *P. aeruginosa* can cause UTIs (Yeruham et al., 2006) and both *E. coli* and *P. aeruginosa* frequently cause mastitis (Banerjee et al., 2017; Lavon et al., 2019). Despite their association with UTIs in bovines, publicly available bacterial genomic sequences from bovine urinary samples are lacking. While there is one bovine urinary isolate genome for *E. coli* (E. coli MOD1-EC6169; Accession number GCF_002536995) in GenBank, there is no further metadata regarding the symptom status of the animal. There are no publicly available genomic sequences in GenBank for urinary *P. aeruginosa* isolates from cattle. Thus, the study conducted here provides genomic insights into this understudied niche.

The isolates presented here, however, are not associated with disease or symptoms. *E. coli* is a known inhabitant of the vagina of healthy Nelore and Gyr cattle (Laguardia-Nascimento et al., 2015; Giannattasio-Ferraz et al., 2019); the same can now be said for the UT. This finding echoes recent studies investigating *E. coli* in the female UT: *E. coli* persists in the human female UT of healthy individuals and is not genetically distinct from strains that cause UTI (Garretto et al., 2020; Price et al., 2020). Thus, *E. coli* and *P. aeruginosa* may be resident members of the bovine UT microbiota. Observations in the female UT suggest that infections arise from dysbiosis of the healthy microbiota rather than introduction of uropathogens from external sources (Thänert et al., 2019; Garretto et al., 2020). If the same is true for bovines has yet to be established; future isolation and sequencing of uropathogens from bovines with UTIs is needed.

The five *E. coli* strains isolated from the UT of healthy Gyr heifers in the present work all encode for motility genes. Motility has been associated with colonization (Lane et al., 2007) suggesting that these isolates have the potential for causing infection. The five genomes are all representatives of the B1 phylotype. *E. coli* strains belonging to this phylotype have been isolated from the bladders of women with UTI and urge urinary incontinence (Garretto et al., 2020) as well as commensal strains from the human GI tract (Massot et al., 2016; Foster-Nyarko et al., 2021) and bovine GI tract (Arimizu et al., 2019). In comparison with other bovine isolates, the bovine urinary isolates are most similar to each other (Supplementary Figure 1), and when compared to human urinary isolates, the core genomes of the five bovine urinary *E. coli* strains most closely resemble each other (Figure 1). However, when the complete genomes are examined, similarity to human urinary isolates and bovine vaginal isolates was observed (Supplementary Table 4). Prior studies found that uropathogens are frequently introduced from the vaginal tract (Yeruham et al., 2006). This suggests that the bovine UT and vaginal microbiomes may be interconnected; a similar observation has been made for the female human UT and vaginal microbiomes (Thomas-White K. et al., 2018).

While the *E. coli* isolates from this study are not associated with UT symptoms, they could emerge as pathogens for humans. In a large genomic comparison of *E. coli* isolated from the bovine and human GI tracts, commensal bovine strains were phylogenetically distinct from commensal human strains (Arimizu et al., 2019). However, the phylogenetic analysis found that ∼80% of the Shiga-toxin producing *E. coli* (STEC) and enteropathogenic *E. coli* (EPEC) strains isolated from humans belong to the bovine lineage (Arimizu et al., 2019). Cattle are a reservoir of STEC, and a prominent concern in Brazil—the world’s largest producer and exporter of beef. Nevertheless, recent surveys find that STEC monitoring is inconsistent across the country (Castro et al., 2019; de Assis et al., 2021). While none of the bovine urinary *E. coli* isolates encode for the Shiga-toxin (or any other toxins) (Supplementary Table 3), further characterization of UT *E. coli* is needed to ascertain if the UT, like the bovine GI, microbiota can serve as a reservoir for STEC strains. Furthermore, several other bacterial species have been associated with zoonosis through urine of infected cattle [see review (McDaniel et al., 2014)].

While most *P. aeruginosa* research in cattle is focused on the species’ association with mastitis bovine (Mbindyo et al., 2020), here we have shown that *P. aeruginosa* may be a resident member of the bovine urinary microbiota. As the animals from which our *P. aeruginosa* strains were isolated did not exhibit any UT symptoms, we cannot speculate as to if *P. aeruginosa* is a commensal. It is worth noting that within the human urinary microbiome, *P. aeruginosa* has been predominantly identified in individuals with lower UT symptoms (Thomas-White K. et al., 2018). Analyses of the core genome and whole genome revealed that the bovine urinary *P. aeruginosa* strains share significant similarity to human urinary isolates. Interestingly, whole genome analyses did not find genomic similarities between *P. aeruginosa* strains from the bovine UT and bovine mastitis isolates (Supplementary Table 4). This suggests that the UT is not likely a source for mastitis. Furthermore, it leads us to posit niche-specific adaptation of *P. aeruginosa* in bovine rather than host-specific adaptation, analogous to what has been observed for cystic fibrosis strains (Dettman and Kassen, 2021).

Interestingly, all 4 bovine urinary *P. aeruginosa* strains encode genes associated with antibiotic resistance [*catB7*, *bla*OXA_50_, *bla*PAO, *fos*A and *aph*(3’-11b)] widely reported in *P. aeruginosa* of clinical isolates, which can be a concern for human public health. Also, these 4 isolates’ genomes belong to the O5 serotype, which is related to multidrug-resistance (MDR) and epidemic virulent strains in humans (Hu et al., 2021). While fecal contamination and milk purity have been the focus of monitoring agricultural products (Garcia et al., 2019; Gray and Mazet, 2020), additional studies of the bovine UT microbiota are needed to assess if the UT could also be a possible route for spreading uropathogens among animals and/or humans.

Examination of the prophage sequences harbored by human and bovine *E. coli* and *P. aeruginosa* isolates from the UT reveals that both bacterial species carry a diverse array of viral species, including both tailed and filamentous (family: Inoviridae) phages. Prior work has found that the bacteria of the human bladder is dominated by lysogens (Miller-Ensminger et al., 2018). In contrast to human urinary isolates, the bovine isolates only harbor a single prophage each. Prophage sequences have been identified in other species isolated from the bovine UT, e.g., *Vagococcus fluvialis* (Giannattasio-Ferraz et al., 2021b). Further investigation into prophage load within bovine urinary bacteria is needed in order to speak more fully about the prevalence of lysogeny within this niche.

While our focus is on the prophages within the bovine isolates, our examination of prophages in human urinary strains reveals that the prophages harbored by the bovine strains are not unique to the bovine UT, i.e., phages of the bovine microbiota are not distinct from other mammalian microbiota. Our knowledge of phages within the bovine microbiome is primarily from rumen fluid and fecal samples [(Hallewell et al., 2014; Sazinas et al., 2019); see review (Gilbert et al., 2020)]. Previously we isolated 6 coliphages from the female bladder microbiota (Malki et al., 2016) and all 6 had greatest sequence similarity to phages isolated from cattle slurry (Smith et al., 2015), leading us to hypothesize that related urinary phages may persist and/or be shared between mammals. This is further supported here: the bovine urinary coliphage and *P. aeruginosa* prophage exhibit greatest sequence similarity to prophages harbored by distantly related bacterial strains isolated from humans (Supplementary Figures 2, 3).

The 5 *E. coli* bovine isolates all contained the same prophage as did the 4 bovine *P. aeruginosa* isolates. One possible explanation is that bacterial host and phage populations can circulate between animals of the same herd. This is consistent with an early study of the bovine rumen virome which found cohabitating animals had more similar viromes (Ross et al., 2013) and some viruses may be conserved across animals (Solden et al., 2018). Given the significant sequence similarity in the bovine urinary isolates and their harbored prophages, we posit that these strains – although isolated from different animals—are the same clonal strain. Comparison of urinary microbiota and/or isolates from animals from different herds would likely reveal less genomic similarity. To date no bovine urinary microbiomes and very few bovine urinary isolates have been sequenced and characterized.

Focusing on just two species here, we have shown that the UT of healthy cattle contains uropathogens. While these two species are associated with disease in both humans and cattle, they are not associated with disease or symptoms in the animals from which they were isolated. Similarity between the bacterial strains supports the current working hypothesis that bacteria can be transmitted between animals. We also found that the prophages encoded by these bacteria exhibit significant sequence similarity to prophages encoded by distantly related strains from the human UT. Future studies of the bovine urinary microbiota are needed to ascertain if *E. coli* and *P. aeruginosa* are resident members of this niche and/or possible sources for emerging pathogens in humans.

## Data Availability Statement

The genome assemblies and raw sequencing reads for the data presented in this article are publicly available in the NCBI Assembly Database (www.ncbi.nlm.nih.gov/assembly) and NCBI Short Read Archive Database (www.ncbi.nlm.nih.gov/sra), respectively. Accession numbers can be found in Table 1.

## Ethics Statement

The animal study was reviewed and approved by Ethics Committee in Animal Experimentation of the Universidade Federal de Minas Gerais, Brazil CEUA/UFMG – 40/2019. Written informed consent was obtained from the owners for the participation of their animals in this study.

## Author Contributions

SG-F, CP, and EFB-S conceived and designed the experiments and wrote the manuscript. SG-F, AE, LM, AO, VG, and CQ performed the experiments. SG-F, AE, and CP analyzed the data. AE and CP produced visualizations. CP and EFB-S contributed reagents, materials, and analysis tools. All authors reviewed the manuscript.

## Conflict of Interest

The authors declare that the research was conducted in the absence of any commercial or financial relationships that could be construed as a potential conflict of interest.

## Publisher’s Note

All claims expressed in this article are solely those of the authors and do not necessarily represent those of their affiliated organizations, or those of the publisher, the editors and the reviewers. Any product that may be evaluated in this article, or claim that may be made by its manufacturer, is not guaranteed or endorsed by the publisher.

## Footnotes

1
https://github.com/najoshi/sickle

2
https://sourceforge.net/projects/bbmap/

3
https://github.com/aene1/bioi_scripts/blob/main/Phaster_network.py
