# Supplementary figures and images for: Escherichia coli and Pseudomonas aeruginosa Isolated From Urine of Healthy Bovine Have Potential as Emerging Human and Bovine Pathogens
Source: Front Microbiol. 2022 Mar 7;13:764760. doi: 10.3389/fmicb.2022.764760 (PMC8940275; doi:10.3389/fmicb.2022.764760)

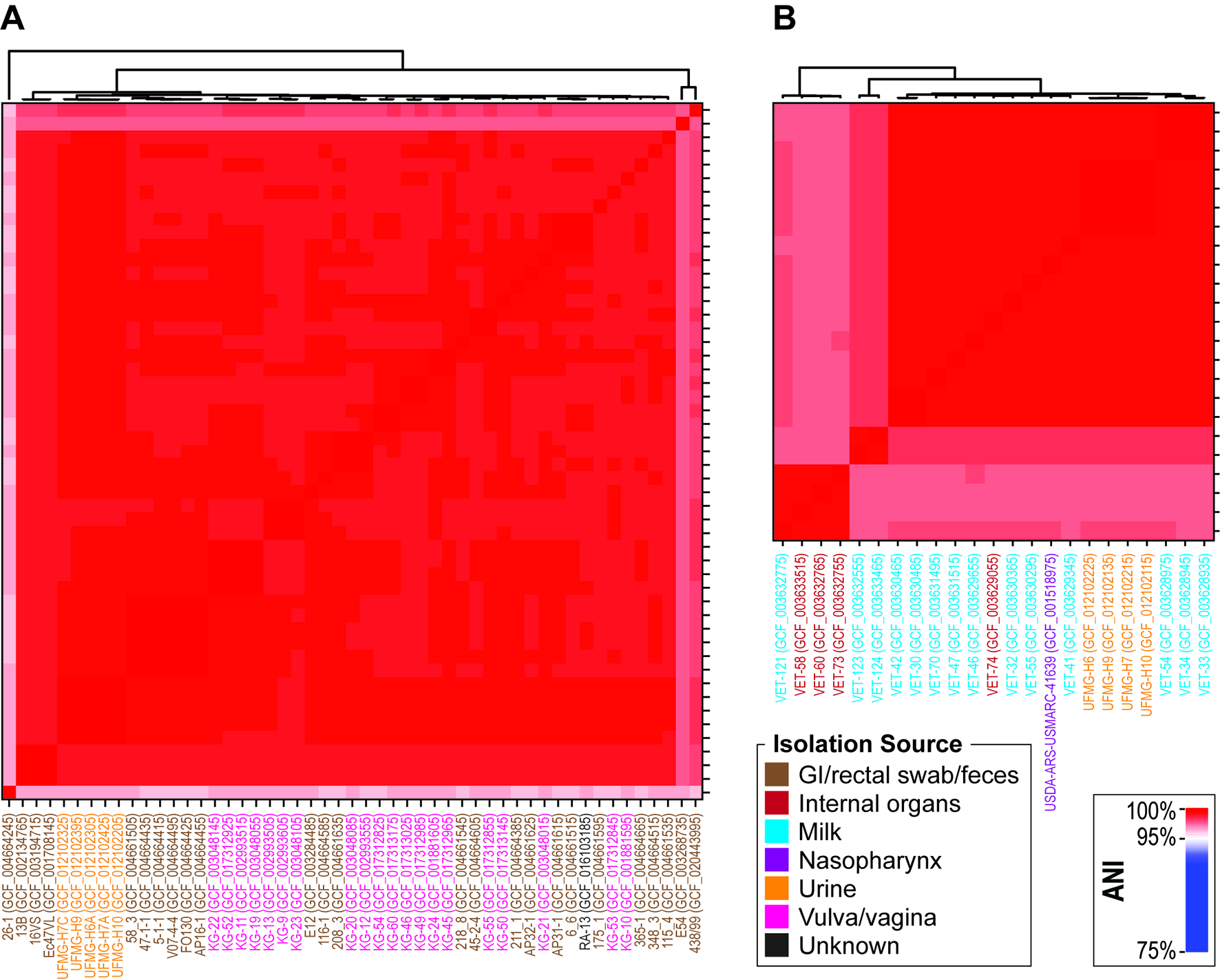

Supplement: Supplementary file 5 [file Image_1.TIF]

A

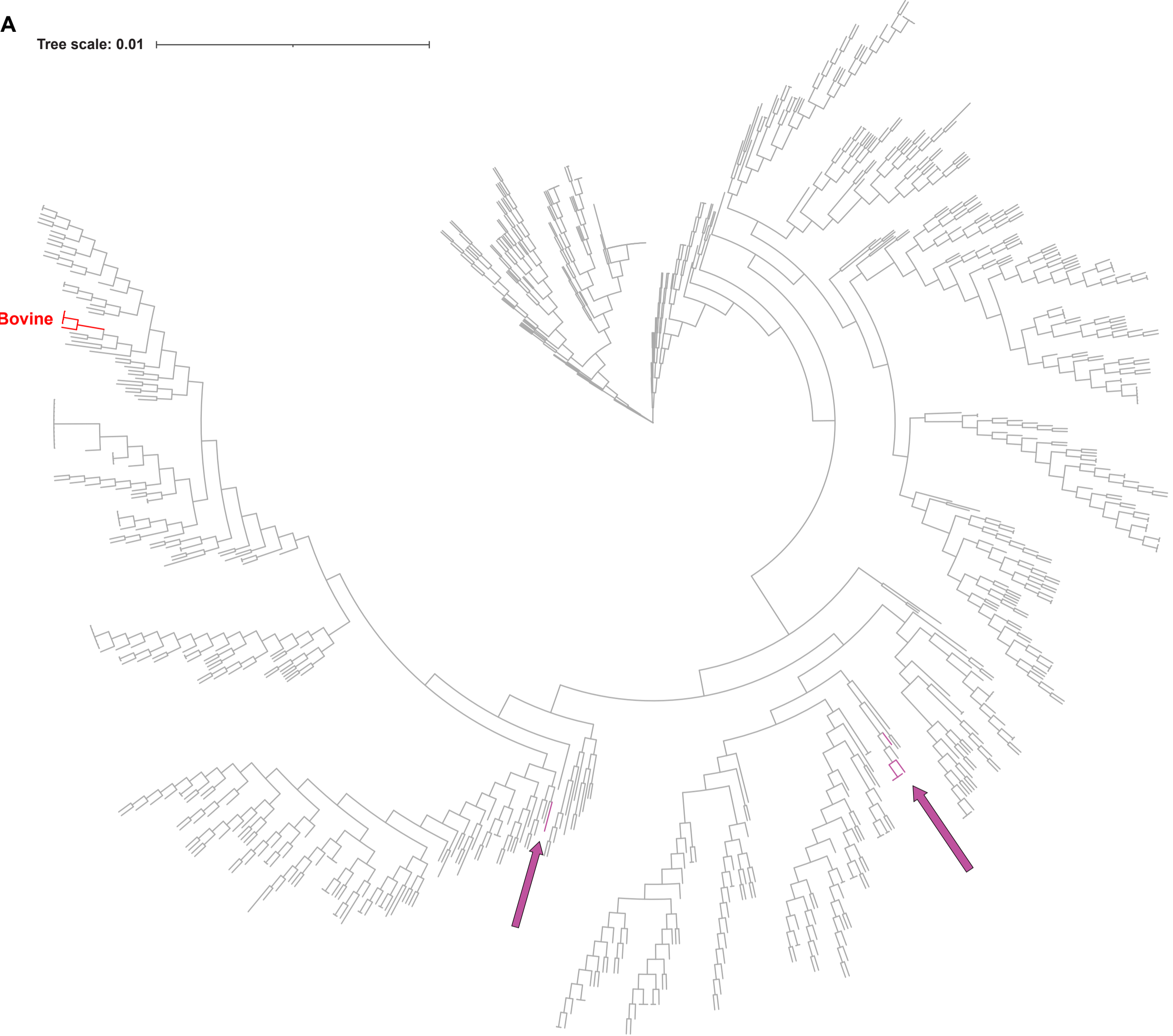

B

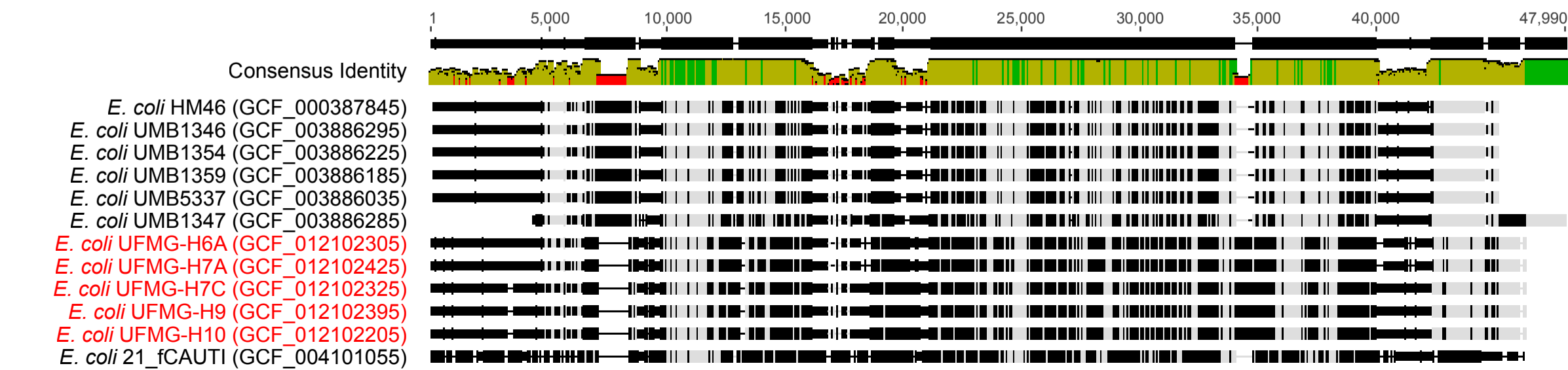

Supplement: Supplementary file 6 [file Image_2.PDF]

A

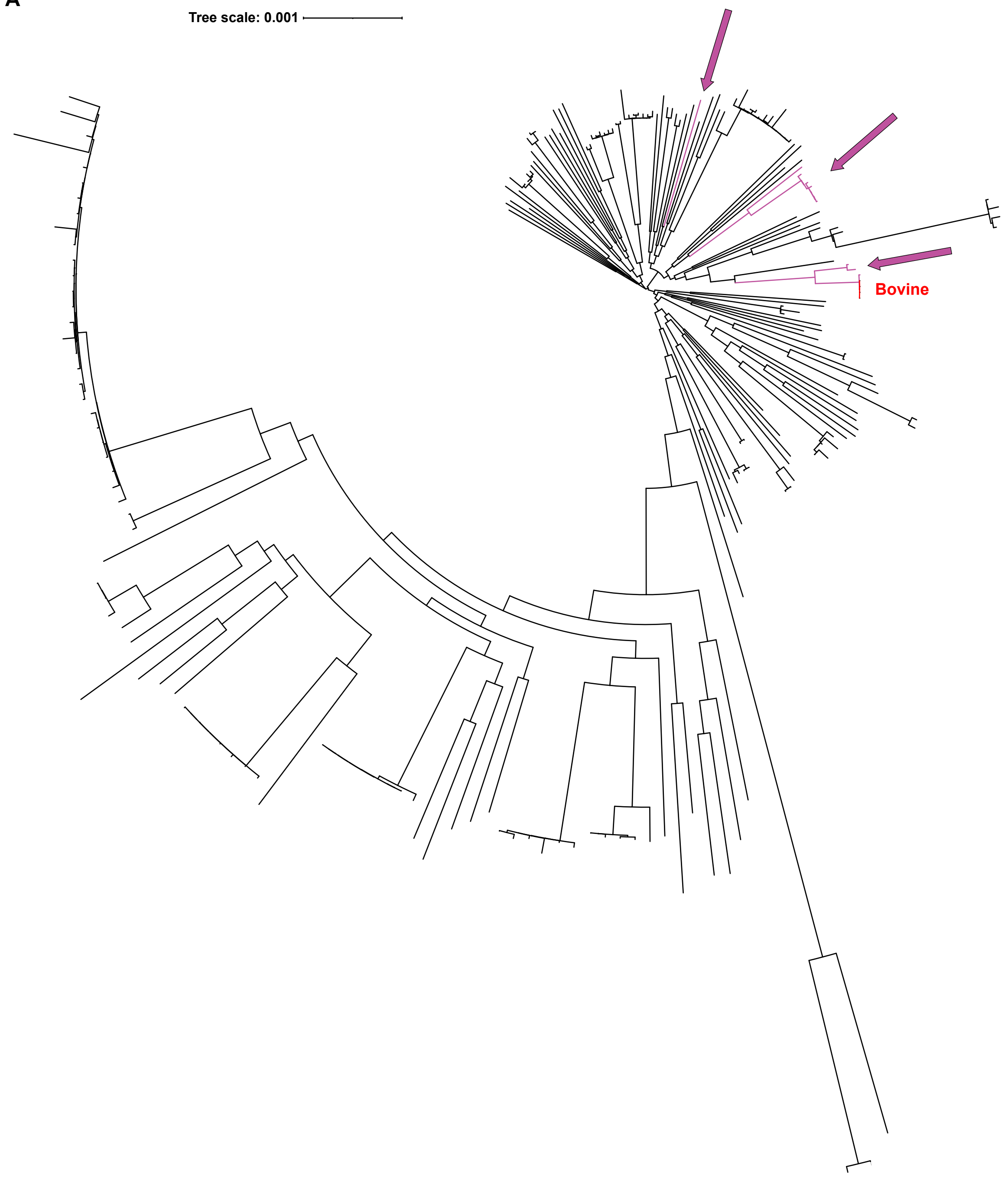

B

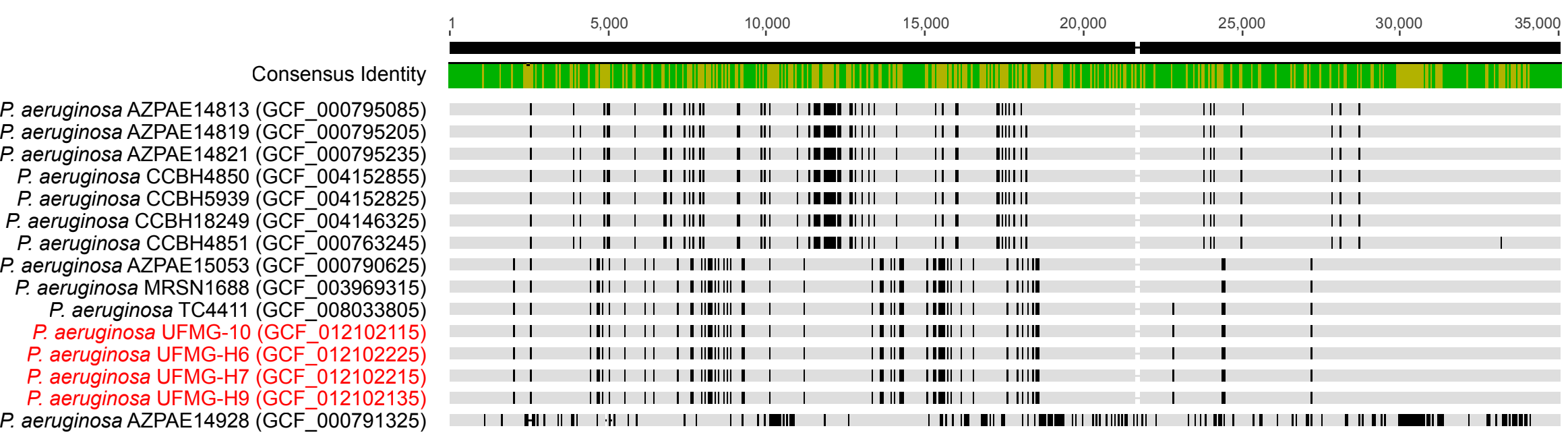

Supplement: Supplementary file 7 [file Image_3.PDF]
